# Selected Indicators Used in Cephalometric Analysis and Their Predictive Value in Defining Sagittal Discrepancy Malocclusions: A Comparative Study

**DOI:** 10.3390/jcm14103429

**Published:** 2025-05-14

**Authors:** Jacek Kotuła, Krzysztof Kotuła, Małgorzata Kotarska, Joanna Lis, Beata Kawala, Michał Sarul, Anna Ewa Kuc

**Affiliations:** 1 Department of Dentofacial Orthopedics and Orthodontics, Wroclaw Medical University, Krakowska 26, 50-425 Wroclaw, Poland; malgorzata.cieplucha@gmail.com (M.K.); joanna.lis@umed.wroc.pl (J.L.); beata.kawala@umed.wroc.pl (B.K.); dental.star@wp.pl (A.E.K.); 2 Faculty of Medicine, Pomeranian Medical University, 70-204 Szczecin, Poland; krzyskotula@gmail.com; 3 Department of Integrated Dentistry, Wroclaw Medical University, Krakowska 26, 50-425 Wroclaw, Poland; michal.sarul@umw.edu.pl

**Keywords:** cephalometry, orthodontics, predictive value

## Abstract

**Background:** In the coming years, the lateral cephalogram will remain, in many cases, the preferred diagnostic tool for planning and reliably assessing the results of orthodontic treatment. The aim of this study was to compare the predictive value and agreement of angular and linear measurements, in terms of precision, in assessing the sagittal discrepancy of the maxillary bases. **Methods:** The study group consisted of 270 cephalometric images of patients aged 12–18 years of both sexes. **Results:** Skeletal classification was performed by comparing the values of the obtained measurements between the corresponding ranges of the compared analyses. Assuming the current standard in class II assessments, based on the ANB angle value, the values closest to this standard with *p* = 0.001 and OR (95%CI) were, in order, the Yen angle analysis (sensitivity 0.994), Tau (0.884), Sar (0.881), W (0.874) and Wits measurements (0.824). The highest predictive value was determined in comparison to the ANB value in the following order: Sar (0.688), W (0.687), Tau (0.709) and Wits (0.707). In the assessment of class III defects with similar assumptions, the closest to the analysis of the ANB angle in the assessment of sagittal discrepancy were, in the order of analysis, Wits (sensitivity 0.737), Sar (0.725), Tau (0.708), W (0.692) and Yen (0.575). The highest predictive value was determined in comparison to ANB in the following order: Yen (0.947), W (0.903), Sar (0.890) and Tau (0.886). **Conclusions:** The presented study confirms the possibility of using the new cephalometric measurements Tau, Yen, Sar and W as a supplement to the previous measurements of the ANB and Wits angles in the assessment of sagittal discrepancy. The results also indicate a higher sensitivity and specificity of the W and Sar angles in comparison to ANB and Wits.

## 1. Introduction

Cephalometric analysis is one of the most important diagnostic tools in orthodontics [1,2,3,4,5,6,7,8,9,10,11,12,13,14,15,16,17,18,19,20,21,22,23]. It allows for the assessment of the mutual relations between the bone masses of the maxillary bases, alveolar processes, teeth and surrounding soft tissues. In addition to assessing the origin of the malocclusion—skeletal or dental—we can use it to predict the direction and rate of growth, assess facial esthetics and compare the results obtained during orthodontic treatment [23,24,25,26,27,28,29,30,31]. In addition to assessing the origin of the malocclusion, the lateral cephalogram will remain, in many cases, the preferred tool for precise diagnostics, planning and the reliable assessment of the results of orthodontic treatment [1,2,3,4,5,6,7,8,9,10,11,12,13,14,15,16,17,18,19,20,21,22,23,24,25,26,27,28]. Since the independent use of the first cephalometric analyses on two-dimensional head radiographs by Broadbent and Hofrath in 1931, new analyses have been systematically created to assess the sagittal relations of the maxillary bases [8,15,16,17,18,19,20]. Properly performed cephalometric analysis is particularly important in cases of borderline skeletal defects, when tooth extraction is considered for orthodontic indications or when complex orthodontic surgical treatment is planned [4,5,11,12,28,29,30]. The reliability of the high evaluation of three-dimensional cephalograms, despite initially promising an overall improvement in the interobserver and intraobserver reproducibility of some landmarks compared to 2D (two-dimensional imaging) imaging [31,32,33,34,35,36,37,38], was perceived less enthusiastically in later studies [39,40,41,42,43] due to both the three-dimensional image accuracy and the possibility of assessing the occurrence of skeletal asymmetry. These controversies, as well as the aspect of patient exposure to higher doses of ionizing radiation, should limit the performance of 3D (three-dimensional imaging) examinations to cases of complex defects, such as deeply impacted canines or for planning maxillofacial surgery [32,39,40,41,42,43,44,45,46,47]. Therefore, assuming that in the coming years the lateral cephalogram will remain, in many cases, the preferred diagnostic tool, not only for treatment planning, but also for reliable evaluation of the results of even orthognathic surgeries [42,43,44,45,46,47,48,49,50,51,52,53], then confirming the reliability of its analysis methods in the contemporary patient population is the responsibility of researchers. This is all the more so because when planning orthodontic and orthognathic treatment, as well as providing therapy for obstructive sleep apnea (OSA) and sleep disordered breathing (SDB), it is still necessary to precisely define reference points that assess the mutual position of anatomical structures in the sagittal and vertical dimensions, including the mandibular base, dentoalveolar mass and soft tissues. This precision, despite the dynamic development of medical knowledge and imaging techniques, is subject to multifactorial limitations [4,15]. Theoretically [23,54,55,56,57], they can be overcome by the development of modern technologies, namely artificial intelligence (AI) use in the technical stages of introducing reference points and performing cephalometric measurements, but there has been a long-standing discussion in the literature about the accuracy and repeatability of measurements performed by operators compared to those generated by AI [54,55,56,57,58,59], and this dispute remains unresolved after a systematic review [23].

The systematic search for new analyses is associated with imperfections in finding reference points on which linear and angular measurements are based [5,6,7] and their instability due to growth and orthodontic treatment [5,6,7,11,12,13,16,17,18,19,20,21,22,23]. In order to reduce the occurrence of diagnostic errors based on unstable anatomical points, especially in borderline situations, clinicians try to compare measurements from several analyses. Previous studies on the repeatability and reliability of the diagnosis of skeletal sagittal discrepancy were mainly based on reference points for determining the ANB angle, the values of which were individualized in the Segner and Hasund method, Wits measurement, AF_BF, APDI and Beta angle [29,30,31,60]. In the meantime, many cephalometric analyses have been created, the aim of which is to minimize errors in determining routinely used reference points or to identify others, which are less susceptible to changes related to growth or orthodontic treatment [5,6,7,17,19,20,21,22,23,24]. Such features—as shown by a systematic review conducted in 2022 by Kotuła et al. [5]—have points necessary to determine at least the Yen [16], Tau [12], W [18] and Sar [19] angles. In studies conducted by Kotuła et al. [6,7] in relation to the reliability of the measurements of the ANB, Tau, Yen, Sar and W angles, the degree of repeatability and reproducibility of determining new parameters for the assessment of sagittal discrepancy were assessed. The aim of this study was to compare the predictive value and agreement of angular and linear measurements in terms of comparing the precision of their use for the assessment of the sagittal discrepancy of the maxillary bases. For this purpose, the strength of the relationship was examined between two different analyses used to classify patients into the same skeletal classes. The sensitivity (effectiveness) of each of the analyses (ANB, Wits, Tau, Yen, Sar and W), which is defined by the percentage of correctly identified individuals with the corresponding skeletal class, was also assessed.

**Figure 1:**
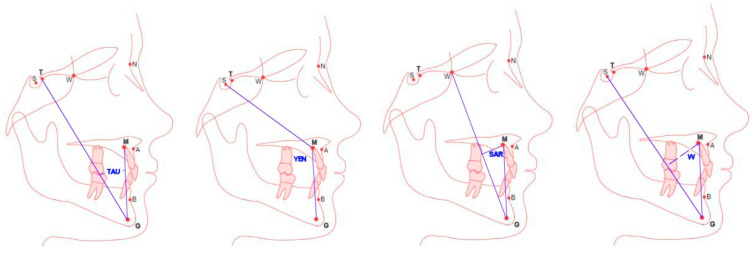
The cephalometric measurements: Tau, Yen, Sar and W angles.

Figure 1 discusses the principles of determining anthropometric points defining the angular measurements Tau, Yen, Sar and W, assessing the sagittal discrepancy of maxillary bases in cephalometric diagnostics. The Yen angle is measured at point M between the arms SM and MG. The Tau angle is measured at point G between the arms TG and GM. The Sar angle is measured at point M between the MG arms and perpendicular to the WG segment derived from point M. The W angle is measured at point M between the MG arms and perpendicular to the SG segment derived from point M. The locations of new anthropometric points were defined as follows: point T is the highest point at the junction of the anterior wall of the pituitary fossa and the sellar tubercle; point M is the construction point representing the center of the largest circle, tangent to the frontal, upper and palatine surfaces of the maxilla; point G is the focus of the circle tangent to the inner, frontal, posterior and lower edges of the mandibular–bifid symphysis.

## 2. Material and Methods

### 2.1. Ethical Considerations

This study was approved by the Bioethics Committee of the District Medical Chamber in Zielona Góra (decision 01/173/2023 of 6 March 2023). Informed consent, in accordance with the guidelines of the Declaration of Helsinki [61], was obtained from all participants taking part in the study and/or their guardians. Patients and their parents were informed about the purpose of the study, the value of the obtained results, the principles of personal data protection of the GDPR (General Data Protection Regulation), the voluntary nature of participation in the study and the possibility of withdrawal and revocation of informed consent at any stage of the project implementation.

### 2.2. Study Conditions and Inclusion and Exclusion Criteria

The study participants were patients of the Department of Maxillofacial Orthopedics and Orthodontics of the Medical University of Wrocław, in whom the diagnostic process was initiated before planning orthodontic treatment.

#### Inclusion and Exclusion Criteria from the Study

The following inclusion criteria were established:

(a) patients of Caucasian race aged 12–18 years
(b) patients prior to orthodontic treatment
(c) patients with various skeletal categories of class I, II and III
(d) patients characterized by various angles of maxillary base inclination
(e) generally healthy patients
(f) patients without developmental defects
(g) patients without symptoms of untreated dental caries and periodontal disease.

The exclusion criteria for the study were as follows:

(a) patients aged 0–12 and over 18 years
(b) patients of a race other than Caucasian
(c) patients who had started or completed orthodontic treatment
(d) asymmetry interpreted as a discrepancy between the contours of the right and left side of the mandibular body greater than 8 mm
(e) a projection error or incorrect contrast preventing the identification of reference points
(f) shifts in bilateral anatomical structures relative to each other
(g) patients with congenital defects
(h) patients with disease burdens, including systemic diseases
(i) patients with a high frequency of caries, periodontal disease or cavities after losing teeth, identified in a clinical examination.

A lateral X-ray of the skull in habitual occlusion was performed in each of the patients qualified for the study. The parallelism of the Frankfurt plane to the ground and the perpendicularity of the beam to the sagittal plane were maintained. After taking the cephalometric image, a visual evaluation of the X-ray image was performed.

### 2.3. Methods

The sample size was calculated using G*Power software version 3.1.9.7 (University of Cologne, Germany) based on initial measurements with a significance level of *p =* 0.05, d = 0.5, a 95% confidence interval and 85% power. A total of 270 digital lateral cephalograms made in a 2D technique were qualified for evaluation. Each cephalogram was recorded as the last 6 digits of the patient’s PESEL number (11-digit identification number, assigned to every person residing in Poland, used for unambiguous identification). The radiographs with the PESEL numbers recorded in this way were assigned numbers from 1 to 270 in any order. The PESEL numbers, together with the corresponding numbers from 1 to 270, were placed in an Excel (Microsoft 2023, Seattle, WA, USA) spreadsheet.

The analysis of all radiographs was performed based on reference points entered into the computer in the Ortodoncja V.9.0 program (Ortobajt^®^, Wrocław, Poland) using NEC Multisync EA 244 WMI monitors of a high quality certified by the laboratory (NEC, Tokyo, Japan). Monitor specifications: diagonal 24 inches, resolution 1920 × 1200, pixel size 0.270 mm, viewing angle 178° vertically and 178° horizontally and contrast 1000:1. The identification of landmarks was performed manually on digital images using a mouse-controlled cursor in the Orthodontics V.9.0 program. Each cephalometric image was entered into the program and initially calibrated (J.K.) according to the measuring ruler placed in the calibration window of the program at the level of 3 cm. The images prepared in this way after initial calibration were sent to all participants of the study. The results were recorded in an Excel spreadsheet (Microsoft, Seattle, WA, USA).

After obtaining radiographs coded and calibrated by the leader (J.K.) and entered directly into the Orthodontics program, the study participants performed 2 series of analyses at 7-day intervals. Each analysis consisted of entering 14 anatomical points: A, N, B, S, T, W, M, G, UIS, UIA, IIS, IIA, U6 and L6.

Once completed, the coded examinations were sent back to the leader and entered into Excel spreadsheets, for each radiograph they were then grouped and statistically analyzed. Cephalometric analyses of the measurements ANB, Wits, Tau, Yen, Sar and W were performed twice with an interval of 7 days by each of the 22 orthodontists. A total of 1848 measurements were obtained, measurements made after determining 25,872 anthropometric points.

Then, statistical analysis of the predictive value and the Fleiss Kappa index was performed. The statistical module V.13.3 (Tibco Software Inc., Palo Alto, CA, USA) was used for statistical analysis.

### 2.4. Statistical Analysis

The analysis, which used the statistical module V.13.3 (Tibco Software Inc., Palo Alto, CA, USA), included the following:

1. An assessment of the precision of individual cephalometric analyses relative to each other;
2. A comparison of the reliability of classifying patients into individual skeletal classes using individual cephalometric analyses.

### 2.5. Analysis of the Predictive Value and the Fleiss Kappa Index

PPV (positive predictive value). To assess the precision of individual cephalometric analyses, the predictive value was used at the significance level of *p* < 0.001, with a CI = 95% strength of the relationship between the compared parameters. The PPV is a measure of the predictive quality of a statistical test. A positive PPV value was considered to be an equal value of the proportion of true positive results of the correct skeletal classifications of two measurements among all positive results. A high PPV value indicates the high precision of the test, which results in a similar classification to the appropriate skeletal class using each of the two compared analyses. PPV does not depend solely on its characteristics. The predictive value also depends on the frequency of the positive state in the population (in the case of orthodontics, on the occurrence of a specific skeletal class). PPV therefore corresponds to the precision index [62]. At the same time, the positive predictive value allows for the expression of the probability that a statistically significant result signals the detection of a true phenomenon of diagnostic convergence of the compared parameters. Insufficiently high PPV values indicate that the studies have an average low power and test uncertain hypotheses.

Another method used to compare two classification systems to the appropriate skeletal classes is the Fleiss’ Kappa test [63,64]. The Fleiss’ Kappa coefficient of agreement is a statistic used to assess the agreement between several methods that classify elements into a number of categories. The value of this coefficient can range from −1 to 1, where values close to 1 indicate a high agreement between raters, and values close to 0 indicate agreement at a random level. The interpretation of the Fleiss’ Kappa value is often based on scales created by different researchers.

One of the more popular proposals, presented by Landis and Koch [64], is as follows:

- <0.00: No agreement
- 0.00–0.20: Poor agreement
- 0.21–0.40: Moderate agreement
- 0.41–0.60: Moderate agreement
- 0.61–0.80: Considerable agreement
- 0.81–1.00: Almost perfect agreement.

## 3. Results

The precision of the individual measurements for assessing the sagittal incongruence of the maxillary bases was assessed based on their characteristics and the frequency of patients being classified into a specific skeletal class.

In order to assess the chance of classifying patients into skeletal class II using individual analyses, a comparison was made of the proportion of patients in class II in relation to patients in classes I and III. Skeletal classification was made by comparing the values of the obtained measurements between the appropriate ranges of the compared analyses at *p* < 0.001 and OR = 95% CI (*p* < 0.001—significance level; CI = 95% strength of the relationship between the compared parameters; OR—odds ratio; CI—confidence level).

OR is the odds ratio and is a statistical measure used to assess the strength of the association between two events, such as exposure to a factor and the occurrence of a specific outcome (e.g., specific skeletal classes). Sens. is the sensitivity of the test, or a measure of the effectiveness of a diagnostic test in detecting the appropriate skeletal class in individuals who truly represent the pattern of the appropriate skeletal class. The sensitivity of the test indicates what percentage of individuals who have a specific skeletal configuration will be correctly identified by the test as individuals who have that skeletal configuration exactly.

PPV—positive predictive value is an indicator that describes how likely it is that a person with a positive test result actually has what the test indicates, and is characterized by a specific skeletal class or condition that the test diagnoses. PPV determines how reliable a positive test result is in real-world conditions [64].

Contingency Table 1, Table 2, Table 3, Table 4, Table 5, Table 6, Table 7, Table 8, Table 9 and Table 10 summarize the results by assessing the relationship between the ANB angle assessment, as the current standard in assessing sagittal incongruence, and classifications based on the assessment of the remaining Wits parameters and the angles Tau, Yen, Sar and W. Table 1, Table 2, Table 3, Table 4 and Table 5 evaluate the comparison of individual parameters with the ANB angle analysis in relation to determining distoclusion. Table 6, Table 7, Table 8, Table 9 and Table 10 evaluate the comparison of individual parameters with the ANB angle analysis in determining mesiocclusion. The evaluation of the predictive value and sensitivity of the test was conducted at the significance level of *p* < 0.001 with a confidence interval of 95%.

Table 1 compares the relationship between the ANB angle measurement and Tau in those belonging to skeletal class II. The test sensitivity of 0.860 indicates a high convergence of the assessment. The predictive value ppv (0.702) indicates that in over 70% of cases, compared to the ANB angle, the occurrence of distoclusion can be confirmed using the Tau index.

Assessing the odds ratio (OR), it can be stated that in patients with a TAU angle > 33.3°, the chance of distoclusion (skeletal class II determined on the basis of the ANB angle) is 28 times higher compared to patients with Tau ≤ 33.3° (OR = 28.0).

Table 2 compares the relationship between the ANB angle measurement and Yen in the classification of skeletal class II. The high sensitivity of the test of 0.994 indicates the excellent convergence of the assessment. The predictive value of ppv (0.506) indicates that in more than 50% of cases, compared to the ANB angle, the occurrence of distoclusion can be confirmed using the Yen angle analysis.

Assessing the odds ratio (OR), it can be stated that in patients with a Yen angle < 129°, the chance of distoclusion (skeletal class II determined on the basis of the ANB angle) is 169 times higher compared to patients with Yen ≥ 129.0° (OR = 169.0).

Table 3 compares the relationship between the ANB angle measurement and W in the classification of skeletal class II. The test sensitivity of 0.874 indicates the high convergence of the assessment. The predictive value ppv (0.687) indicates that in almost 70% of cases, in comparison with the ANB angle, the occurrence of distoclusion can be confirmed using the W index.

Assessing the odds ratio (OR), it can be stated that in patients with a W angle < 54.1°, the chance of distoclusion (skeletal class II determined based on the ANB angle) is 28.3 times higher compared to patients with W ≥ 54.1° (OR = 28.3).

Table 4 compares the relationship between the ANB angle measurement and Sar in the classification of skeletal class II. The test sensitivity of 0.881 indicates the high convergence of the assessment. The predictive value ppv (0.688) indicates that in almost 70% of cases, compared to the ANB angle, the occurrence of distoclusion can be confirmed using the Sar angle analysis.

Assessing the odds ratio (OR), it can be stated that in patients with a Sar angle < 56.6°, the chance of distoclusion (skeletal class II determined on the basis of the ANB angle) is 29.9 times higher compared to patients with Sar ≥ 56.6° (OR = 29.9).

Table 5 compares the relationship between the ANB angle measurement and the Wits measurement in the classification of skeletal class II. The test sensitivity of 0.824 indicates the high convergence of the assessment. The predictive value ppv (0.707) indicates that in over 70% of cases, in comparison with the ANB angle, the occurrence of distoclusion can be confirmed using the Wits index.

Assessing the odds ratio (OR), it can be stated that in patients with a Wits measurement >1.0, the chance of distoclusion (skeletal class II determined on the basis of the ANB angle) is 23 times higher in comparison with patients with Wits ≤ 1.0 mm (OR = 23.0).

The numerical values refer to the analyses of individual measurements in relation to ANB—Tau/Yen/Sar/W/Wits in the assessment of distoclusion:

- Sensitivity = 86/99.4/88.1/87.4/82.4%.
- Positive predictive value = 70.2/50.6/68.8/68.7/70.7%.
- Odds ratio (OR—Likelihood Ratio) = 28.0/169/29.9/28.3/23

Based on the analysis of the diagnostic accuracy parameters of the test, their highest values should be stated in the assessment of the YEN angle (sensitivity 0.994), Tau angle (sensitivity 0.884), SAR (sensitivity 0.881) and W (0.874).

Comparing the ORs, it can be assumed that in the case of finding distoclusion using the ANB angle, its confirmation in the analysis of the remaining parameters in the appropriate ranges for each measurement corresponding to skeletal class II is on average over twenty times higher than when classified as class I or III. The most appropriate in this assessment is the Yen angle, and the least sensitive is the Wits measurement.

Assuming that the “gold standard” is the classification of distoclusion based on the ANB angle value, the closest to this standard is the classification based on the YEN angle value (sensitivity 0.994), Tau angle (sensitivity 0.884), SAR (sensitivity 0.881) and W (0.874).

Table 6 compares the relationship between the ANB angle measurement and the Tau angle in the classification of skeletal class III. The test sensitivity of 0.708 indicates the good convergence of the assessment. The predictive value of ppv (0.884) indicates that in over 88% of cases, compared to the ANB angle, the occurrence of mesiocclusion can be confirmed using the Tau angle.

Assessing the odds ratio (OR), it can be stated that in patients with Tau angle values < 29.9°, the chance of mesiocclusion (skeletal class III determined based on the ANB angle) is 31.7 times higher compared to patients with a Tau angle value ≥ 29.9° (OR = 31.7).

Table 7 compares the relationship between the ANB angle measurement and the Yen angle in those belonging to skeletal class III. The test sensitivity of 0.575 indicates the good convergence of the assessment. The predictive value ppv (0.947) indicates that in over 94% of cases, compared to the ANB angle, the occurrence of mesiocclusion can be confirmed using the Yen angle.

Assessing the odds ratio (OR), it can be stated that in patients with Yen angle values > 127. 2°, the chance of mesiocclusion (skeletal class III determined on the basis of the ANB angle) is 53.4 times higher compared to patients with a Yen angle value ≤ 127.2° (OR = 53.4).

Table 8 compares the relationship between the ANB angle measurement and the W angle in those belonging to skeletal class III. The test sensitivity of 0.692 indicates the good convergence of the assessment. The predictive value ppv (0.903) indicates that in over 90% of cases, compared to the ANB angle, the occurrence of mesiocclusion can be confirmed using the W angle.

Assessing the odds ratio (OR), it can be stated that in patients with W angle values > 57.5°, the chance of mesiocclusion (skeletal class III determined on the basis of the ANB angle) is 37 times higher compared to patients with the W angle value ≤ 57.5° (OR = 37.0).

Table 9 compares the relationship between the ANB angle measurement and the Sar angle in those belonging to skeletal class III. The test sensitivity of 0.725 indicates the good convergence of the assessment. The predictive value ppv (0.890) indicates that in over 89% of cases, compared to the ANB angle, the occurrence of mesiocclusion can be confirmed using the Sar angle.

Assessing the odds ratio (OR), it can be stated that in patients with Sar angle values > 59.6°, the chance of mesiocclusion (skeletal class III determined on the basis of the ANB angle) is 35.4 times higher compared to patients with the Sar angle value ≤ 59.6° (OR = 35.4).

Table 10 compares the relationship between the ANB angle measurement and the Wits measurement in the classification of skeletal class III. The test sensitivity of 0.737 indicates the good convergence of the assessment. The predictive value of ppv (0.896) indicates that in almost 90% of cases, compared to the ANB angle, the occurrence of mesiocclusion can be confirmed using the Wits measurement.

Assessing the odds ratio (OR), it can be stated that in patients with Wits measurement values < −0.8 mm, the chance of mesiocclusion (skeletal class III determined based on the ANB angle) is 40 times higher compared to patients with a Wits value ≥ −0.8 mm (OR = 40).

The numerical values refer to the analyses of individual measurements in relation to ANB—Tau/Yen/Sar/W/Wits in the assessment of mesiocclusion:

- Sensitivity = 70.8/57.5/72.5/87.4/73.7%.
- Positive predictive value = 88.4/94.7/89.0/68.7/89.6%.
- Odds ratio (OR—Likelihood Ratio) = 31.7/53.4/35.4/28.3/40.0.

Based on the analysis of the diagnostic accuracy parameters of the test, its highest value should be stated in the assessment of the W angle (sensitivity 0.874), Wits (sensitivity 0.737), SAR (sensitivity 0.725), Tau (0.708) and Yen (sensitivity 0.575).

Comparing the ORs, it can be assumed that in the case of mesiocclusion detected using the ANB angle, its confirmation in the analysis of the remaining parameters in the appropriate ranges for each measurement corresponding to skeletal class III is on average 28–53 times higher than when classified as class I or III. The most appropriate in this assessment is the Yen angle, and the least sensitive is the W angle.

Assuming that the standard for classifying mesiocclusion is the ANB angle value, the closest to this standard is the classification based on the Wits index (sensitivity 0.737), Sar angle (sensitivity 0.725), Tau angle (sensitivity 0.708), W angle (sensitivity 0.692) and Yen (0.575).

Another way to compare two methods of classifying radiographs into one appropriate skeletal class may be the Fleiss Kappa index (Table 11).

Moderate agreement was observed between the standard, i.e., the classification based on the ANB angle value and the classification based on the Tau, W and Sar angle values and the Wits parameter (Table 11). Moderate agreement was also observed with the Yen angle values.

## 4. Discussion

The assessment of sagittal discrepancy in cephalometric analysis is a fundamental task during diagnosis and orthodontic treatment planning [1,2,3,4,5,6,7,8,9,10,11,12,13,14,15,16,17,18,19,20,21,22,23,24,33,35,36,37,38,39,40,41,42,43,44,45,46]. Clinicians are systematically looking for a reliable measurement that will demonstrate greater accuracy in assessing class II and/or III malocclusions than the standard ANB angle analysis [3,4,5,6,7,11,13,17,18,19,20,21,22,23]. The search for a reliable measurement focuses on assessing the repeatability and reproducibility of new reference points [4,5,6] or angular or linear measurements [5,6].

In the presented work, the predictive values of the parameters for assessing sagittal incongruence were determined, which were included in the systematic review from 2022 [4].

In assessing the possibilities of confirming the classification of individual radiographs to skeletal class II or III, the new angular measurements were correlated with the ANB and Wits angle measurements considered to be standard.

The assessment of the value of the analyses of posterior defects in comparison to the ANB classification indicated that the Tau and Wits parameters had the highest comparative value. With good test sensitivity at the level of 86% and 82.4%, they allowed for the correct classification of patients to class II in over 70% of cases, identical to ANB. At the same time, the odds ratio of correct classification to class II indicated a 28- and 23-times greater chance, respectively, than classification to class I or III. The Yen angle had the highest chance of correct classification with a 169-times greater probability of correct classification.

The evaluation of the value of the analyses of anterior defects in comparison to the ANB classification indicated that the Yen and Wits parameters had the highest comparative value. With satisfactory test sensitivities of 57.5% and 73.7%, over 94.7% and 89.6% allowed for the correct classification of patients into class III, identical to ANB. At the same time, the odds ratio of correct classification into class III indicated a 53.4- and 40-times greater chance than classification into class I or II.

In the literature to date, authors have not encountered a predictive assessment of the possibility of reliably replacing the assessment of one analysis with the results of another, nor with an assessment of the test sensitivity and test reliability in real conditions.

Previous studies by Neel et al. [16] and Kumar et al. [18] indicated the higher reliability and stability of the Yen angle analysis based on anatomical points G and M as being more reliable, with 100% test sensitivity in relation to ANB in the assessment of sagittal incongruence. Gupta et al. [12] also assessed the Tau angle and indicated its excellent repeatability and reproducibility in determining sagittal abnormalities. The studies by Kotuła et al. [5,6] were not as optimistic for the Yen and Tau measurements.

Also, the values of Tau, Yen, Sar and W measurements in the assessment of sagittal discrepancy included in the publications [5,6,7] indicated the higher reliability of the ANB angle analysis in the assessment of sagittal discrepancy of the maxillary bases. The results obtained in the current studies, especially in relation to the Yen angle, indicated the occurrence of a specific phenomenon. The high sensitivity of the Yen angle (99.4%) indicated a high probability of correctly classifying a patient with skeletal class II to this class. However, the low Kappa agreement (0.345) in comparison with the ANB angle indicated a high discrepancy in classifying malocclusions to class II using each of these parameters. Such a discrepancy may indicate the imperfection of the ANB angle as an appropriate reference point. We are well aware that the ANB angle, previously considered as a standard in the assessment of sagittal incongruence, is not ideal. Its value depends on the points A, N and B, which are subject to growth changes [5,6,7,11,12,16,17,18,19,20]. The different assessment of the ANB angle value is also facilitated by irregularities in the positioning of points S and N in relation to the configuration of the skull base, asymmetries and mandibular rotations. Previous studies have indicated discrepancies in the repeatability of determining points A and B in relation to the OX axis of the Cartesian system, especially in correlation with different values of the angles of the maxillary bases. Previous studies [6,7] have shown the greater stability of points M and G in relation to A, N and B. A greater deviation in point G and M in relation to the OY axis of the Cartesian system has been shown [6]. It should also be considered correct that there is little or even no dependence on the length of the skull base when assessing the Yen angle. Thanks to this, the error of class II assessment associated with the retroposition of the mandible with the correct position of the maxilla is minimized. The methodological differences in the measurement of both angles justify the possibility of there being low agreement between the possibility of classifying defects based on them to the same skeletal class with a high sensitivity index of the Yen angle. When assessing the accuracy of cephalometric analysis results, it should be taken into account that these are only additional tests, the assessment of which belongs to the physician. No method is completely free from errors, and in some situations the obtained results may require validation using an alternative method [1,2,5,6,7,16,17,18,19,20,24,25].

Nevertheless, the result of cephalometric analysis may be one of the factors qualifying the patient for a given treatment method.

In the case of class II defects, the patient may be treated functionally or through camouflage. Functional treatment often requires a two-stage procedure and is therefore a longer treatment [33,45,46,51,64]. In addition, the effectiveness of the treatment is largely determined by the patient’s cooperation [51,64]. In turn, treatment through camouflage usually takes less time, but is also not free from specific complications related to the distalization of all teeth or of the anterior maxillary teeth [49,56]. In this situation, a change in measurement by 2 degrees may be one of the factors determining the classification of the patient into a given group.

In the case of class III defects, the early qualification of patients for conservative treatment and prognosis regarding the probability of the need for surgical treatment are particularly important [46,47,48,49]. Also, in the case of the treatment of young borderline patients in whom both camouflage and orthognathic treatment can be considered, the result of cephalometric analysis may be important for the adoption of one or another treatment strategy.

Cephalometry is therefore neither the only nor the most important diagnostic method, but it can have a significant impact on the decisions made by the doctor. In this context, striving for the highest possible accuracy and repeatability of the method is of great importance.

We should also not forget about the indirect influence of cephalometric analysis on the decisions made by orthodontists. Evidence-based medicine is based on the use of research results concerning, among other aspects, the effects of a given therapy. The effects of treatment, and therefore the results of the studies, are often quantified and compared precisely as the results of cephalometric analysis. Any inaccuracy in this area is then multiplied by citing the results of a given study and affects the generally accepted consensus regarding, for example, the effectiveness of a given therapeutic method. In this context, striving for the highest possible accuracy of this analysis may be even more important [5,6,7].

The presented study confirms the possibility of using the new cephalometric measurements Tau, Yen, Sar and W as a supplement to the previous ANB angle and Wits measurement in the assessment of sagittal discrepancies [5,6,7,11,12,16,18,19].

In the assessment of the reliability of individual angular measurements, the ability to position anthropometric points based on the knowledge and experience of orthodontists comes to the fore, because doctors can more easily use the long-standing markers. In order to improve the reliability of measurements, doctors should be trained in the positioning of anatomical points on radiographs. Computer programs should be prepared to assist doctors in visualizing the entered points. Thanks to the development of AI, it is possible to start training artificial intelligence in this area, which could complement the possibilities of mechanically performing cephalometric analysis, leaving its assessment in the hands of an experienced orthodontist [58,59,60].

Due to the limitations of this study, the assessment of individual parameters should be approached critically. Particular caution should be exercised in patients of developmental age and in the analysis of sagittal discrepancy related to the assessment of the base height angle, taking into account the vertical movement of point G with growth.

### 4.1. Limitations

The limitations of this study are the wide age range of patients and the lack of a distinction between genders, which make it difficult to generalize the results to a specific group of patients. Another significant limitation is the lack of possibility to compare images of the same patient taken before and after the growth period. Due to the limitations of this study, the assessment of individual parameters should be approached critically. Particular caution should be exercised in patients of developmental age and in the analysis of sagittal discrepancy related to the assessment of the base height angle, taking into account the vertical movement of the point G with growth.

### 4.2. Future Directions of Research

Further research is justified; in particular, randomized controlled trials (RCTs) can be conducted comparing alternative methods of assessing sagittal discrepancies. Due to the limitations of this study, the possibility of defining groups divided by the gender and age of patients should be considered. An attempt should also be made to assess the predictive value of the classification into individual skeletal classes in relation to the reference to the base angle or the index value in the assessment of vertical discrepancy, taking into account the relationship between sagittal discrepancy and vertical discrepancy.

## 5. Conclusions

The presented study confirms the possibility of using the new cephalometric measurements Tau, Yen, Sar and W as a supplement to the previous measurement of the ANB and Wits angles in the assessment of sagittal discrepancies. The average difference in the repeatability results of all the analyses discussed is small and ranges from −0.09° to 0.03°. Such small average differences between two measurements performed by the same orthodontist allow for the practical use of each of these parameters in the assessment of sagittal discrepancy. However, the new measurements are characterized by a larger error range, which in extreme cases prevents their use in orthodontic diagnostics.

## Figures and Tables

**Table 1:** Number (percentage) of patients in groups differing in their affiliation to skeletal class II based on the ANB angle and TAU angle values.

Skeletal Class II Tau Angle	Skeletal Class II ANB: >4° (Distoclusion)		*p*-Value	OR [95% CI]	Test Quality Indicators
	Yes N = 3933	No N = 7947			
Tau > 33.3°	3384 (86.0)	1434 (18.0)	<0.001	28.0 [25.2–31.2]	Sens. = 0.860
Tau ≤ 33.3°	549 (14.0)	6513 (82.0)		1.00 (ref.)	PPV = 0.702

**Table 2:** Number (percentage) of patients in groups differing in their affiliation to skeletal class II based on the ANB angle and Yen angle values.

Skeletal Class II Yen Angle	Skeletal Class II ANB: >4° (Distoclusion)		*p*-Value	OR [95% CI]	Test Quality Indicators
	Yes N = 3933	No N = 7947			
Yen < 129.0°	3908 (99.4)	3816 (48.0)	<0.001	169 [114–251]	Sens. = 0.994
Yen ≥ 129.0°	25 (0.6)	4131 (52.0)		1.00 (ref.)	PPV = 0.506

**Table 3:** Number (percentage) of patients in groups differing in their affiliation to skeletal class II based on the ANB angle and W angle values.

Skeletal Class II W Angle	Skeletal Class II ANB: >4° (Distoclusion)		*p*-Value	OR [95% CI]	Test Quality Indicators
	Yes N = 3933	No N = 7947			
W < 54.1°	3439 (87.4)	1570 (19.8)	<0.001	28.3 [25.3–31.5]	Sens. = 0.874
W ≥ 54.1°	494 (12.6)	6377 (80.2)		1.00 (ref.)	PPV = 0.687

**Table 4:** Number (percentage) of patients in groups differing in their classification of skeletal class II based on the ANB angle and Sar angle values.

Skeletal Class II Sar Angle	Skeletal Class II ANB: >4° (Distoclusion)		*p*-Value	OR [95% CI]	Test Quality Indicators
	Yes N = 3933	No N = 7947			
Sar < 56.6°	3464 (88.1)	1574 (19.8)	<0.001	29.9 [26.8–33.4]	Sens. = 0.881
Sar ≥ 56.6°	469 (12.6)	6373 (80.2)		1.00 (ref.)	PPV = 0.688

**Table 5:** Number (percentage) of patients in groups differing in their affiliation to skeletal class II based on the ANB angle value and Wits measurement.

Skeletal Class II Wits	Skeletal Class II ANB: >4° (Distoclusion)		*p*-Value	OR [95% CI]	Test Quality Indicators
	Yes N = 3933	No N = 7947			
Wits > 1.0 mm	3240 (82.4)	1341 (16.9)	<0.001	23.0 [20.8–25.5]	Sens. = 0.824
Wits ≤ 1.0 mm	693 (16.6)	6606 (83.1)		1.00 (ref.)	PPV = 0.707

**Table 6:** Number (percentage) of patients in groups differing in their belonging to skeletal class III based on the ANB angle and Tau angle values.

Skeletal Class III Tau Angle	Skeletal Class III ANB: <2° (Mesiocclusion)		*p*-Value	OR [95% CI]	Test Quality Indicators
	Yes N = 5166	No N = 6714			
Tau < 29.9°	3660 (70.8)	478 (7.1)	<0.001	31.7 [28.4–35.4]	Sens. = 0.708
Tau ≥ 29.9°	1506 (29.2)	6236 (92.9)		1.00 (ref.)	PPV = 0.884

**Table 7:** Number (percentage) of patients in groups differing in their affiliation to skeletal class III based on the ANB angle and Yen angle values.

Skeletal Class III Yen Angle	Skeletal Class III ANB: <2° (Mesiocclusion)		*p*-Value	OR [95% CI]	Test Quality Indicators
	Yes N = 5166	No N = 6714			
Yen > 127.2°	2972 (57.5)	166 (2.5)	<0.001	53.4 [45.4–62.9]	Sens. = 0.575
Yen ≤ 127.2°	2194 (42.5)	6548 (97.5)		1.00 (ref.)	PPV = 0.947

**Table 8:** Number (percentage) of patients in groups differing in their affiliation to skeletal class III based on the ANB angle and W angle values.

Skeletal Class III W Angle	Skeletal Class III ANB: <2° (Mesiocclusion)		*p*-Value	OR [95% CI]	Test Quality Indicators
	Yes N = 5166	No N = 6714			
W > 57.5°	3575 (69.2)	384 (5.7)	<0.001	37.0 [32.9–41.7]	Sens. = 0.692
W ≤ 57.5°	1591 (30.8)	6330 (94.3)		1.00 (ref.)	PPV = 0.903

**Table 9:** Number (percentage) of patients in groups differing in their affiliation to skeletal class III based on the ANB angle and Ta Sar angle values.

Skeletal Class III Sar Angle	Skeletal Class III ANB: <2° (Mesiocclusion)		*p*-Value	OR [95% CI]	Test Quality Indicators
	Yes N = 5166	No N = 6714			
Sar > 59.6°	3743 (72.5)	464 (6.9)	<0.001	35.4 [31.7–39.6]	Sens. = 0.725
Sar ≤ 59.6°	1423 (27.5)	6250 (93.1)		1.00 (ref.)	PPV = 0.890

**Table 10:** Number (percentage) of patients in groups differing in their affiliation to skeletal class III based on the ANB angle value and Wits measurement.

Skeletal Class III Wits	Skeletal Class III ANB: <2° (Mesiocclusion)		*p*-Value	OR [95% CI]	Test Quality Indicators
	Yes N = 5166	No N = 6714			
Wits < −0.8 mm	3809 (73.7)	440 (6.5)	<0.001	40.0 [35.7–44.9]	Sens. = 0.737
Wits ≥ −0.8 mm	1357 (26.3)	6274 (93.5)		1.00 (ref.)	PPV = 0.896

**Table 11:** Values of the Fleiss Kappa coefficient of agreement between the ANB classification and the remaining parameters.

	Tau (°)	Yen (°)	W (°)	Sar (°)	Wits (mm)
ANB (°)	0.540	0.345	0.542	0.553	0.546

## Data Availability

Unpublished data from this study are held by the corresponding author and can be made available upon specific and reasonable request.

## Abbreviations

2D	two-dimensional graphics
3D	three-dimensional graphics
AI	artificial intelligence
OSA	obstructive speech apnea
OSD	obstructive sleep disorder
OBS	obstructive sleep breathing
RODO	General Data Protection Regulation
PESEL	the 11-digit identification number assigned to every person residing in Poland and used for unambiguous identification
PPV	positive predictive value
*p*	significance level
OR	odds ratio
CI	confidence level
